# Make it better but don't change anything

**DOI:** 10.1186/1759-4499-1-5

**Published:** 2009-11-19

**Authors:** Jerry M Wright

**Affiliations:** 1 Department of Physiology, Johns Hopkins School of Medicine, 725 N Wolfe St, Baltimore, MD 21205, USA

## Abstract

With massive amounts of data being generated in electronic format, there is a need in basic science laboratories to adopt new methods for tracking and analyzing data. An electronic laboratory notebook (ELN) is not just a replacement for a paper lab notebook, it is a new method of storing and organizing data while maintaining the data entry flexibility and legal recording functions of paper notebooks. Paper notebooks are regarded as highly flexible since the user can configure it to store almost anything that can be written or physically pasted onto the pages. However, data retrieval and data sharing from paper notebooks are labor intensive processes and notebooks can be misplaced, a single point of failure that loses all entries in the volume. Additional features provided by electronic notebooks include searchable indices, data sharing, automatic archiving for security against loss and ease of data duplication. Furthermore, ELNs can be tasked with additional functions not commonly found in paper notebooks such as inventory control. While ELNs have been on the market for some time now, adoption of an ELN in academic basic science laboratories has been lagging. Issues that have restrained development and adoption of ELN in research laboratories are the sheer variety and frequency of changes in protocols with a need for the user to control notebook configuration outside the framework of professional IT staff support. In this commentary, we will look at some of the issues and experiences in academic laboratories that have proved challenging in implementing an electronic lab notebook.

## Introduction

In this issue of Automated Experimentation Axiope introduces eCat, an electronic laboratory notebook (ELN) for basic science research laboratories. Adoption of an ELN in small basic science laboratories has been lagging with only an estimated 4% of academic labs having a system in place [1]. With increasing amounts of data generated in electronic format, lab workers will adopt whatever software that meets their immediate needs such as wikis, blogs and spreadsheets. However, what may work for an individual may not work well with overall laboratory management needs and leads to a proliferation of data archives in a variety of formats that cannot be easily located or integrated.

Advances with web architecture in recent years has made a web based electronic notebook feasible. Browser based notebooks are independent of the computer operating system, permit the lab machine to be upgraded or changed without disturbing the database and allows multiple access points. Web based solutions do not require as robust a desktop computer as would be used at a workstation so cheaper or older computers can be used. And, use of a single database accessible from multiple locations reduces the need for multiple data archives in different formats.

A basic science laboratory is a challenging environment for electronic based record keeping. Critical to needs is a highly customizable electronic record keeping system, akin to having the flexibility of a bound paper notebook, yet be in electronic format so results can be quickly located, analyzed and shared. This additional flexibility needs to be integrated into laboratory procedures while being unobtrusive as possible. Compounding the software issues are time constraints upon the staff, who are often students with a limited period of employment, the need to publish quickly in order to fund the next grant and lack of direct IT support staff. Here we will examine some of the issues involved in creating an ELN suitable for use in a small basic science laboratory.

## Discussion

### Work environment

The basic science lab is a particular challenge because of the nature of the work. It is diverse. If two people are doing the same thing, one of them needs to be doing something else; resources are too limited to permit duplication of effort. Funding is usually tight, sometimes nonexistent. The experimental setup frequently changes. Months can be spent optimizing a process to use for one week getting data needed to prove or disprove a point. As a result, much of the notebook may be dedicated to developing protocols. This makes record tracking even more challenging since computer based software configurations assume a consistency in data format. In academic institutions, staff turnover is guaranteed every time someone graduates. There are significant cultural and educational differences among lab workers which must be taken into account. Generally researchers are highly intelligent, inquisitive, demanding workaholics who have no interest in computer technology; it's just another hammer in the toolbox.

### Implementation

No ELN system will work just by a supervisor demanding electronic reports; the bench scientists must have the proper tools to produce the reports. Successful implementation comes from the top by directive and demonstration. If the lab director approves new experiments by reviewing a western blot image handed off while meeting in the hallway, that image will not make into an ELN. The approval process for an experiment is not part of the final publication so there is little incentive to add data after approval has been granted. Unfortunately for developers, an informal approval process is quite common in small laboratories yet this process may need to be tracked if questions are later raised. Consequently the software must be minimally intrusive and easily set up for recording raw data from analytical equipment. Given the wide variety of scientific file formats, this is no easy task.

Record keeping, data analysis, project tracking and report generation needs of a lab supervisor are quite different from what's needed at the bench. Consequently, the system must meet both demands in order to be successful. At each level the user wants a highly customized product that deals with the project at hand; forcing a change in work habits to meet the needs of the software is a sure way to prevent adoption.

### Terminology

There are a number of barriers to implementation one of which is terminology that may not be clearly defined in the minds of the users or may be defined differently by IT staff and laboratory staff. The first are the distinctions between data, information and knowledge. IT professionals usually make fairly clear distinctions because of computational issues arising when working with the different forms [2]. However a biologist may not make such clear distinctions and not fully comprehend the difficulties inherent in electronically managing the different forms. After all, they can reside side-by-side in a paper notebook. Here the staff scientist is the data integrator but may not be consciously aware of it; transferring that task to an automated system can be a significant challenge. Another issue is the distinction between electronic lab notebooks and laboratory information systems (LIMS). These terms can mean many things to many people depending upon the project and data archiving needs. Fundamentally, an ELN tracks what is done to a sample at a point in a workflow while LIMS tracks sample movement through a work flow; across industry, only about 1/3 of lab scientists are clear about the distinction [3,4]. Ideally, these are separate but well integrated software processes but smaller labs will want to use an ELN to track samples in order to keep costs down.

### Configuration

Electronic notebooks have to be configured for specific projects at hand and multiple features require optimization of each feature. Usually there is a lack of local IT support knowledgeable about laboratory workflow to assist with configuration. This presents the laboratory with the choice of either educating the IT staff on laboratory procedures or adopting a system the lab worker can easily modify to meet the needs of the current project.

### Integration

The ability to integrate the various databases is crucial to large scale collaborative projects. Paper lab notebooks are not well suited for cooperative projects; each one is an island unto itself. For instance, a blood sample is drawn and recorded at a clinic, shipped to a processing lab where mRNA is extracted, processed and run on a microarray, the chip image is scanned and the raw data sent to a statistician for analysis, the analysis is sent to the principal investigator who talks to other experts to fully interpret the results. Each step of the process has data recording and transfer done independently of other stations. Tracking back to the source to find out why a male patient sample has high expression of Barr body generated mRNA is difficult.

### Resources

Publications dealing with technical ELN problems in development, configuration and social interactions affecting implementation are more often found in specialized conference proceedings, vendor white papers or industry magazines not often read in the biological research community or by general IT staff. Articles may be reviewed by an acceptance committee but are usually not subject to the review/revision cycle of peer reviewed scientific publications. The Association for Computing Machinery proceedings offers several resources including the Conference on Human Factors in Computing Systems (CHI) and Computer Supported Cooperative Work (CSCW). Another resource is Scientific Computing which has articles dealing with general ELN and LIMS issues [5,6].

### Summary

An important point is that electronic lab notebooks are really a new form of record keeping. While users demand the interface be easy to use and similar to the standard lab notebook, the additional functions also demanded are far beyond what a pen and ink notebook can deliver. eCat has the promise of being well suited for basic science as well as larger institutional laboratories. The developers have paid attention to areas that cripple implementation initiatives in many basic science laboratories: cost, scaling capabilities, user interface and ability to customize, control of data sharing, image management, version control and electronic signatures. Fortunately the developers have expended considerable effort in optimizing the interface with feedback from real world laboratories. eCat matches the general organizational methods commonly used by many bench researchers. While maintaining the basic functions of a lab notebook with entry points for projects and experiments, the functionality is greatly increased. I believe eCat has had some success in meeting the customer demand that is hardest to accommodate "Make it better but don't change anything".

## Abbreviations

ELN: Electronic Laboratory Notebook; LIMS: Laboratory Information Management System; IT: Information Technology.
